# A rare case of maxillary sinus and buccal space involvement of extramedullary plasmocytoma: Cross-sectional imaging findings and review of the literature

**DOI:** 10.1016/j.radcr.2024.07.171

**Published:** 2024-08-24

**Authors:** Pier Paolo Arcuri, Simonetta Antonelli, Barbara Vavalà, Simona Roccia, Vincenzo Aiello, Marco Rossi, Domenico Laganà

**Affiliations:** a Radiology Unit “De Lellis”, Azienda Ospedaliero-Universitaria “Renato Dulbecco”, Catanzaro 88100, Italy; b Total Quality Unit, Lamezia Terme Hospital, Catanzaro 88100, Italy; c Rheumatology Clinic “Madonna dello Scoglio” Cotronei, Crotone 88836, Italy; d Department of Hematology-Oncology, Azienda Ospedaliero-Universitaria “Renato Dulbecco”, Catanzaro 88100, Italy; e Department of Experimental and Clinical Medicine, ‘Magna Graecia’ Università di Catanzaro, Catanzaro 88100, Italy; f Radiology Unit, Azienda Ospedaliero-Universitaria “Renato Dulbecco”, Catanzaro 88100, Italy

**Keywords:** Extramedullary plasmacytoma, Magnetic resonance imaging, DWI, ADC, PET, Maxillary sinus

## Abstract

Extramedullary plasmacytoma (EMP) belongs to the group of plasma cell neoplasms, which include following entities: multiple myeloma (MM), lymphoplasmacytic lymphoma, solitary plasmacytoma of the bone (SBP) and EMP. Localization in the maxillary sinus with simultaneous involvement of the buccal cavity is rare. Misdiagnosis may lead to inappropriate or delayed management. X-ray, computed tomography (CT) scan, magnetic resonance imaging (MRI) and positron emission tomography/computed tomography (PET/CT) scan provide useful information for diagnosis. Many CT and MRI features are not specific and it is important to find specific imaging characteristics for making differential diagnosis. Our case has shown how, in the context of advanced MRI techniques, DWI is decisive in achieving the correct diagnosis of EMP The peculiarity of this case, in addition to showing the possibility, although rare, of a simultaneous involvement of EMP of the buccal cavity and of the ipsilateral maxillary sinus, presents the behavior of the EMP in various imaging methods, highlighting how diffusion-weighted imaging (DWI) played an important role to suggest the correct diagnosis and differentiating it from squamous cell carcinoma (SCC) and non-Hodgkin lymphoma (NHL).

## Introduction

Plasmocytoma presents various clinical and pathological aspects. They are divided into multiple myeloma (MM), solitary bone plasmacytoma (SBP) when plasma cells malignancies occur as a solitary bone lesion, and extramedullary plasmacytoma (EMP) when they present as a solitary soft tissue lesion [[1], [2], [3]]. Of these, MM occurs most frequently, and SBP and EMP occur rarely, with incidences of 5% and 2%, respectively [4].

Maxillary involvement is rare [2,[5], [6], [7]]. Based on our knowledge, only 15 cases studied with MRI, 12 studied with DWI, none with spectroscopy. A few reports (4 cases) have described a solitary plasmacytoma invading the maxillary sinus and the buccal cavity simultaneously [[5], [6], [7]].

Misdiagnosis may lead to inappropriate or delayed management [8]. X-ray, computed tomography (CT) scan, magnetic resonance imaging (MRI) and Positron Emission Tomography/Computed Tomography (PET/CT) scan provide useful information for diagnosis [[1], [2], [3],^5^,^6^,[8], [9], [10], [11]]. In case we present, diffusion-weighted imaging (DWI) played an important role to suggest the diagnosis of EMP of the left maxillary sinus and buccal space.

### Case presentation

**Fig. 1:**
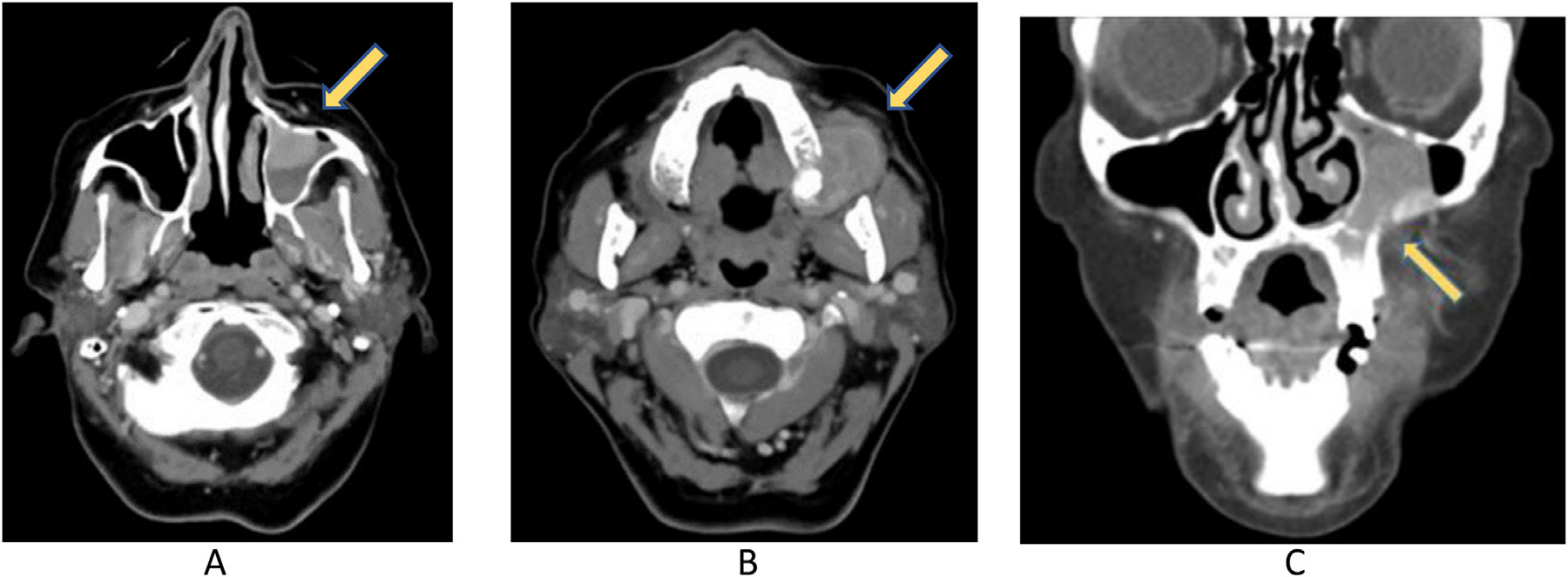
(A-C): enhanced facial CT scan axial plane (A,B) revealed the presence of a soft tissue density lesion in the left maxillary sinus. The lesion showed inhomogeneous contrast-enhancement after iodinate contrast agent administration. Coronal plane (C) image suggested the presence of erosion of the left maxillary sinus floor and extension of the mass to the mouth.

A 54-year-old woman complaining pain in the left maxillary alveolar region and swelling in the left zygomatic area and oral ipsilateral cavity went to our Hospital. These symptoms suggested the presence of a tooth disease or sinusitis, so unenhanced facial CT scan was performed. A soft tissue density lesion was detected in the left maxillary sinus with erosion of the omolateral sinus floor and extension to the mouth. Since there was a suspicion of a neoplastic lesion, CT was completed with iodinated contrast medium, confirming the suspicion by highlighting inhomogeneous enhancement of the same expansive lesion (Fig. 1).

The diagnostic hypotheses essentially focused on: squamous cell carcinoma (SCC), non-Hodgkin lymphoma (NHL) and extramedullary plasmocytoma (EMP).

Therefore, for a better characterization as well as for a more accurate study of the soft tissues surrounding the lesion, we performed a facial MRI scan without and with contrast agent administration (gadolinium) on 1.5 Tesla MRI scanner. To the standard protocol, we have included the DWI sequence and the spectroscopy (MRS) sequence using Two-dimensional Pointed Resolved Spectroscopy (2D PRESS) sequence.

**Fig. 2:**
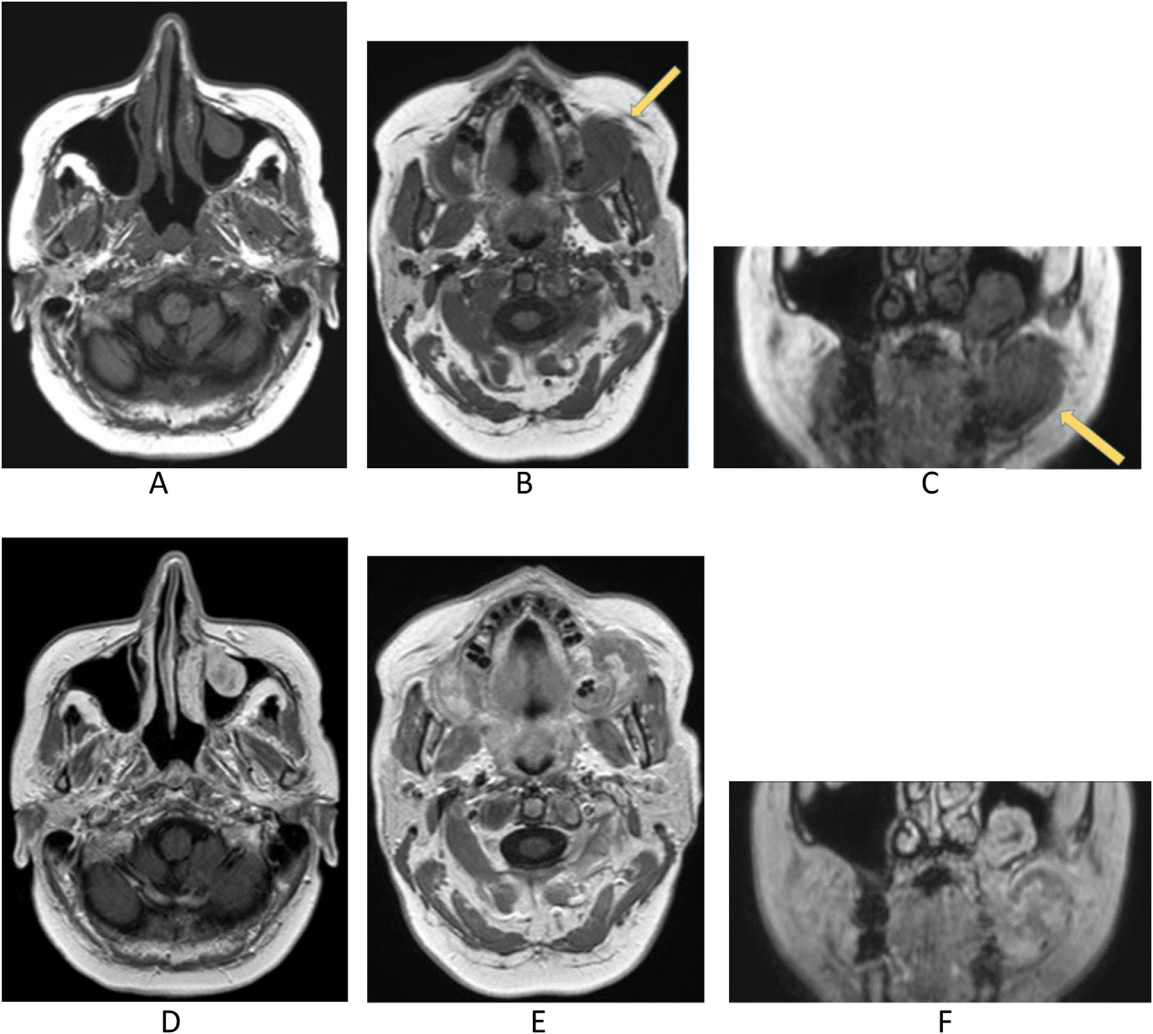
(A-F) MRI T1 SE sequences before contrast medium administration, axial plane (A,B) and coronal plane (C). The lesion showed low-intermediate signal intensity on T1-weighted sequence. The presence of only one lesion extending from the left maxillary sinus to masticator space with erosion of the sinus floor was better evident on the coronal plane (C). After contrast medium administration (D-F) the lesion showed inhomogeneous contrast-enhancement.

**Fig. 3:**
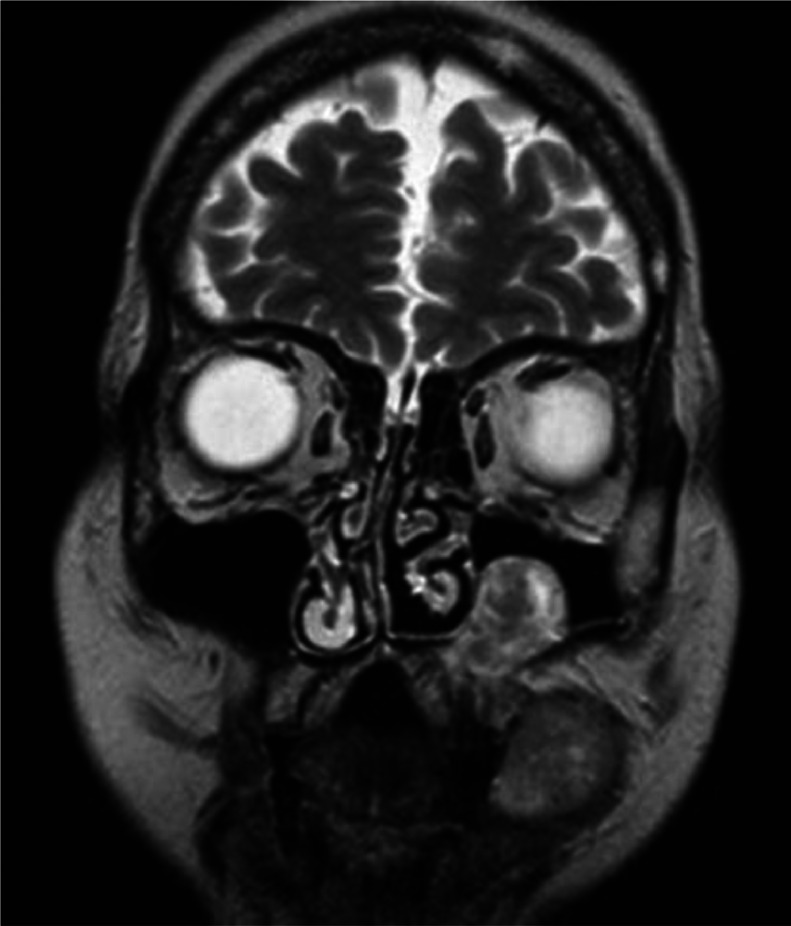
MRI T2 TSE coronal plane. The lesion showed mild T2 signal hyperintensity with erosion of the left alveolar process and homolateral maxillary sinus. Erosion extended inferiorly to the hard palate.

**Fig. 4:**
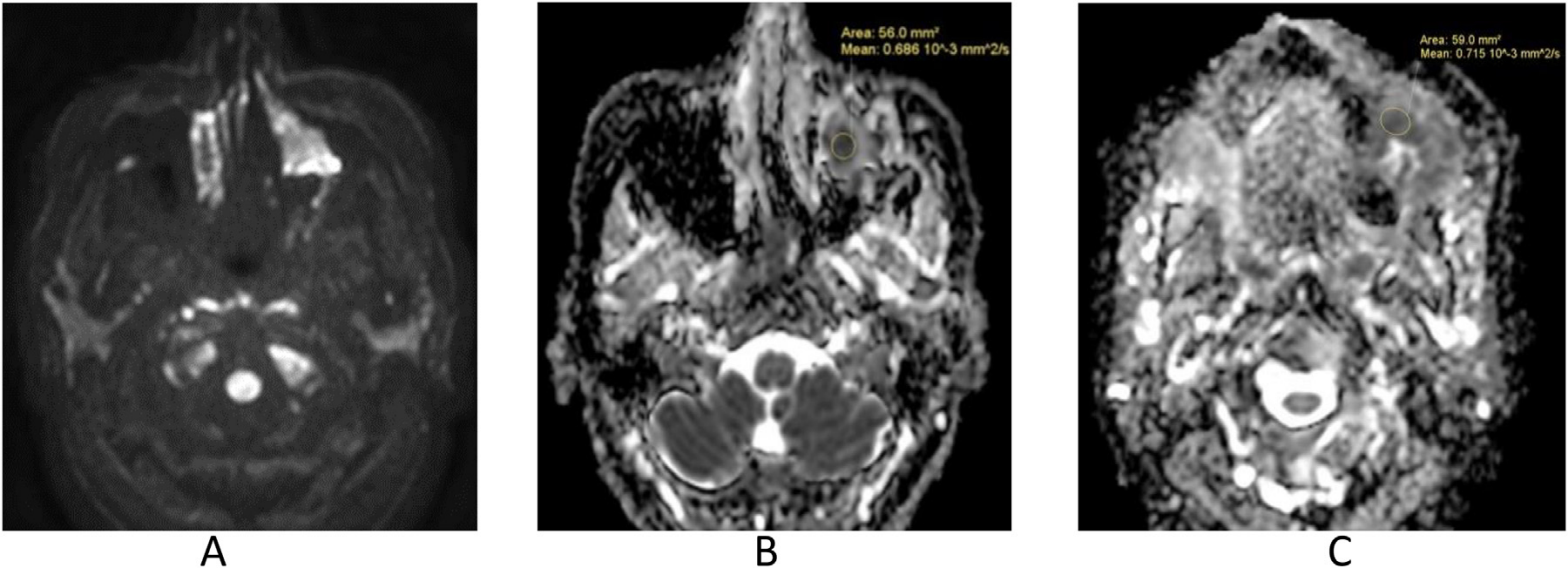
(A-C): DWI (b-value = 800) sequence (A) showed restricted diffusion. ADC map (B, C) revealed a low ADC values of the mass, between 0.6 and 0.7 × 10^−3^ mm^2^/sec: these ADC values suggested the diagnosis of a plasma cells malignancy.

**Fig. 5:**
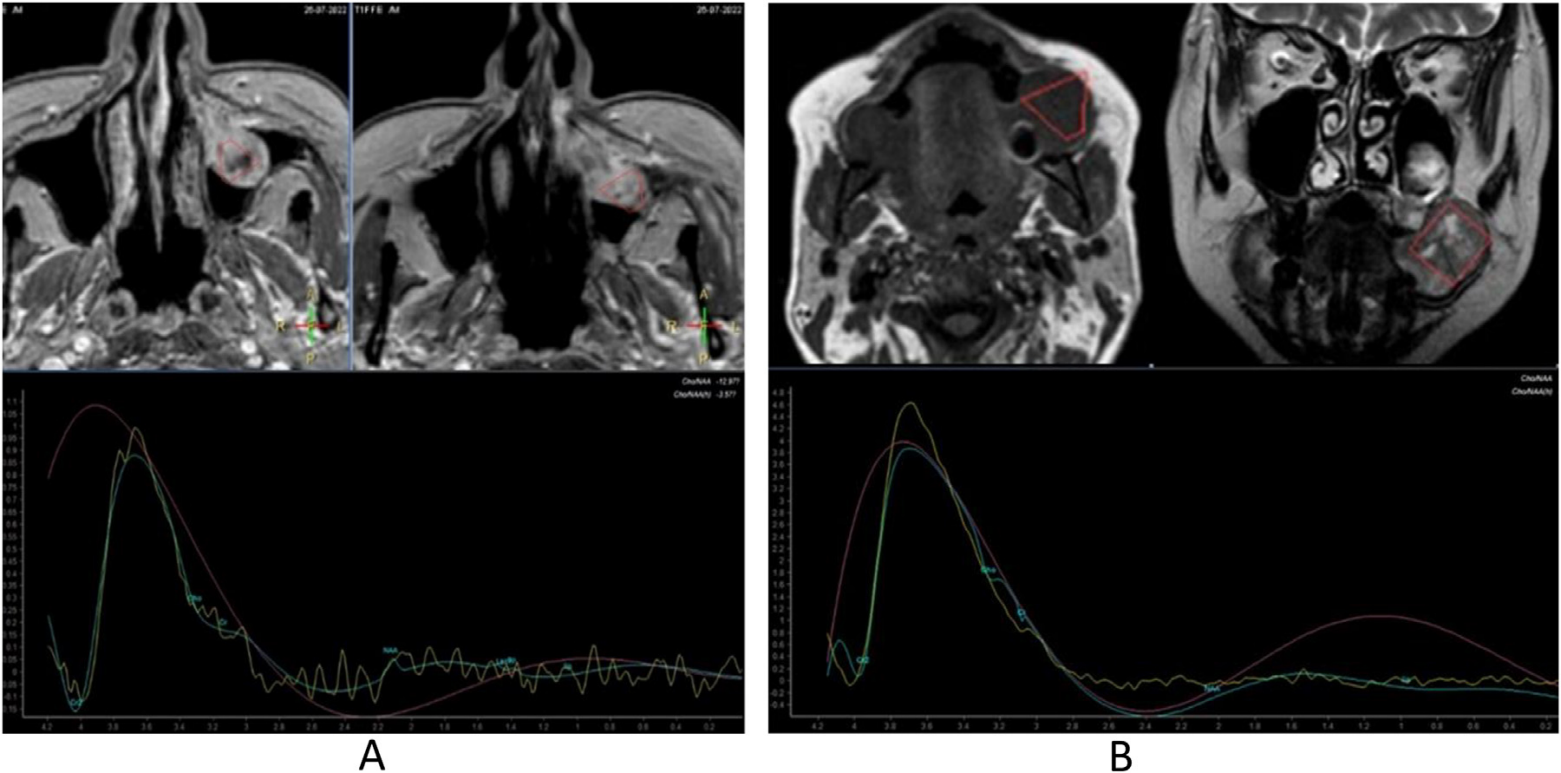
MR spectroscopy features were not specific for tumor.

**Fig. 6:**
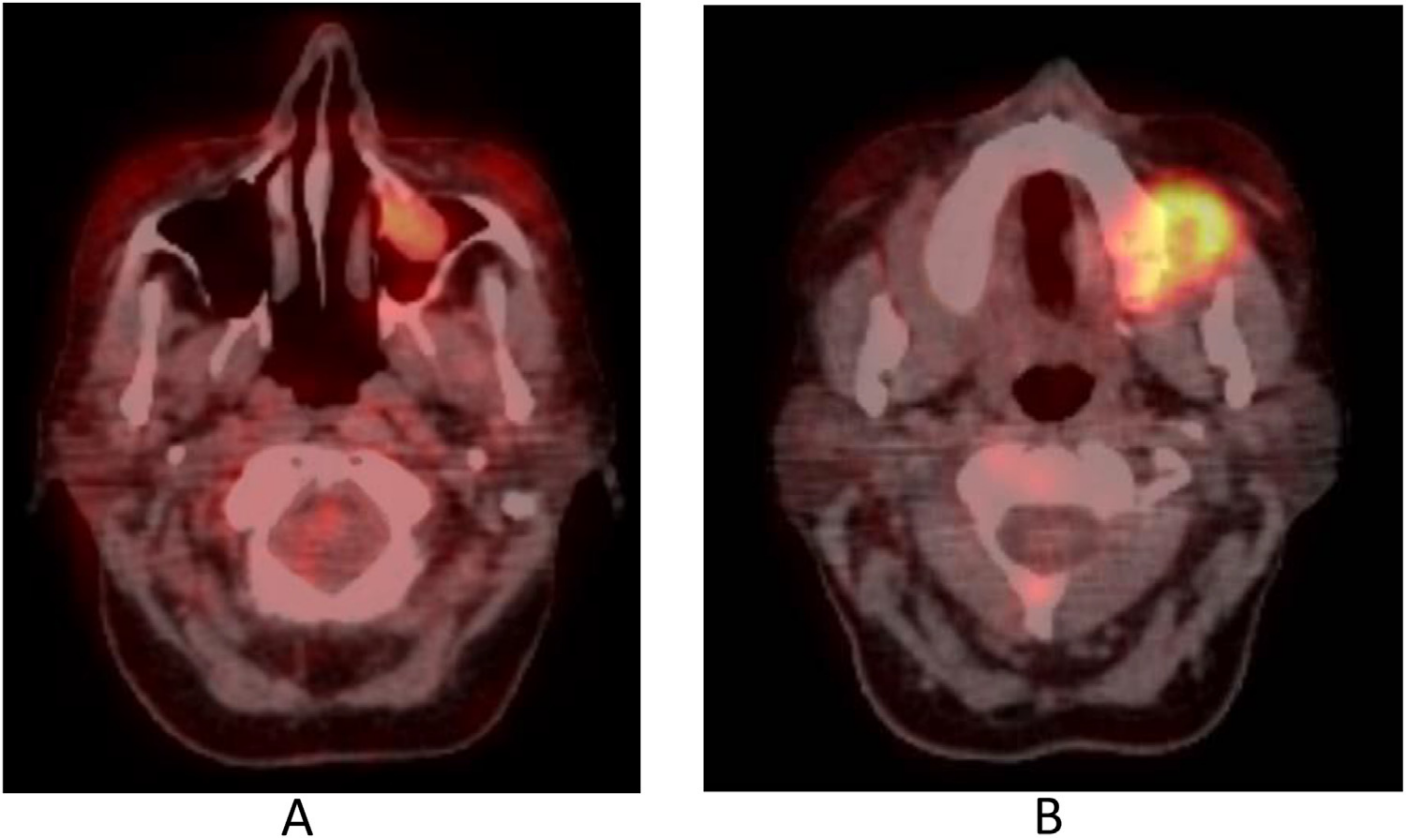
(A, B) PET/CT confirmed the presence of an aggressive lesion showing an increased metabolic activity in the left maxillary sinus (SUV from 5.6 to 15.3).

**Fig. 7:**
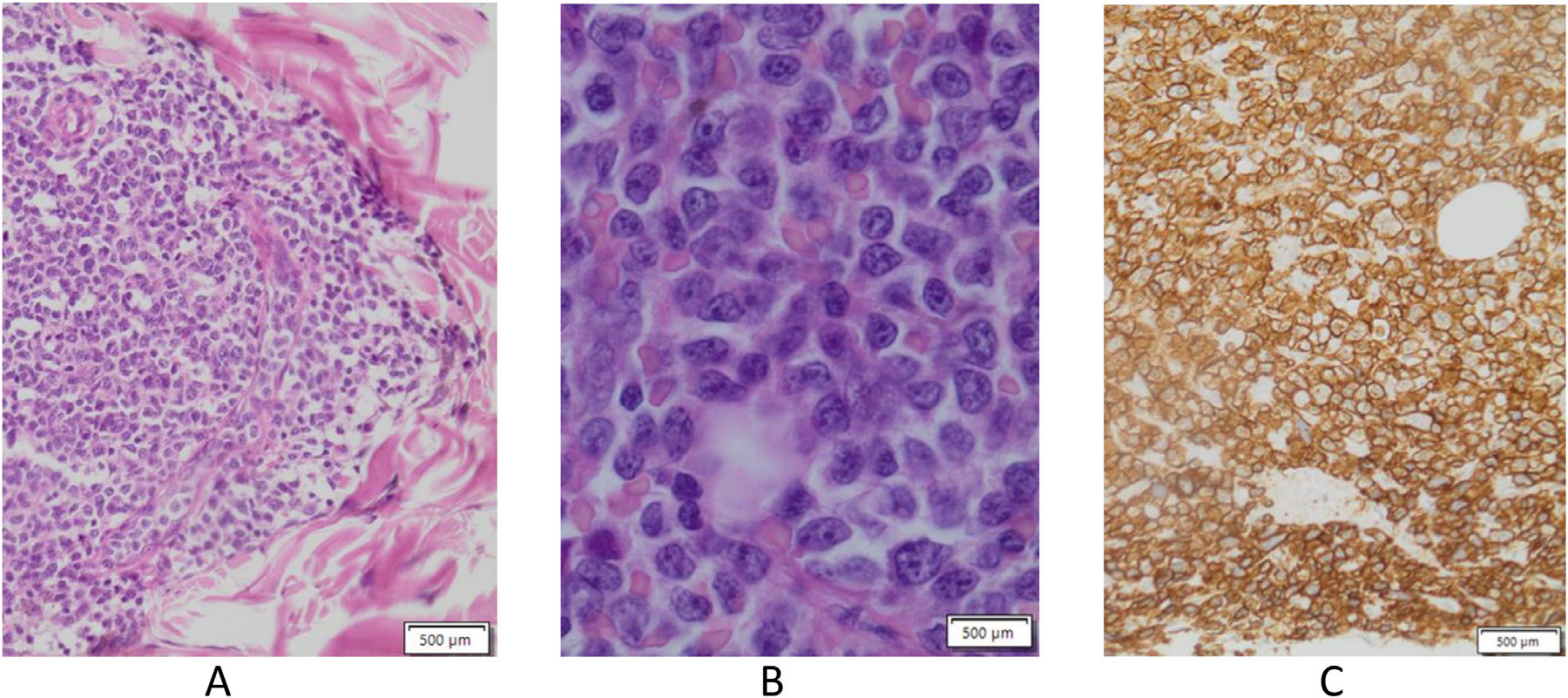
(A-C) Histopathologic findings of the mass. H&E, ×50 (A), ×200 (B) the mass was characterized by the presence of carpet of elements of immature plasma cells, poor cytoplasm, vesicular nucleus, presence of nucleolus. The immune-histochemical study (C) showed CD+ 138 (cytoplasmic and membrane positivity) confirming the diagnosis of multiple myeloma.

MRI (Figs. 2 and 3) confirmed, especially evident on the coronal plane, the heteroplastic expansive lesion occupying the left maxillary sinus and, consequently to the erosion of the floor of the same sinus, the extension to masticator space with erosion of the left alveolar process. The mass showed intermediate signal intensity on T1-weighted sequences, mild T2 signal hyperintensity, inhomogeneous contrast enhancement and restricted diffusion in DWI (Fig. 4). Apparent diffusion coefficients (ADC) were low, between 0.6 and 0.7 × 10^−3^ mm^2^/sec.: these values suggested the diagnosis of a plasma cells malignancy. MR spectroscopy features were not specific for tumor (Figs. 5A and B). After MRI scan, a PET scan was performed to detect other disease localization in the body. PET scan (Fig. 6) confirmed the presence of the aggressive lesion showing an increased metabolic activity (SUV = 10.4) in the left maxillary sinus and masticator ipsilateral space; no other disease localization was detected. To have diagnostic certainty, a biopsy of the lesion was performed: the mass was characterized by the presence of carpet of elements of immature plasma cells (poor cytoplasm, vesicular nucleus, presence of nucleolus). The immune-histochemical study showed CD+ 138 (cytoplasmic and membrane positivity) confirming the diagnosis of multiple myeloma (Fig. 7).

Therefore the patient was transferred to Hematology and Oncology Department for appropriate therapy. As multiple myeloma is sensitive radiation, the main treatment method was radiation therapy, and an irradiation of 40 to 45 Gy or more to the lesion produced the best results for local control without any side effects.

**Fig. 8:**
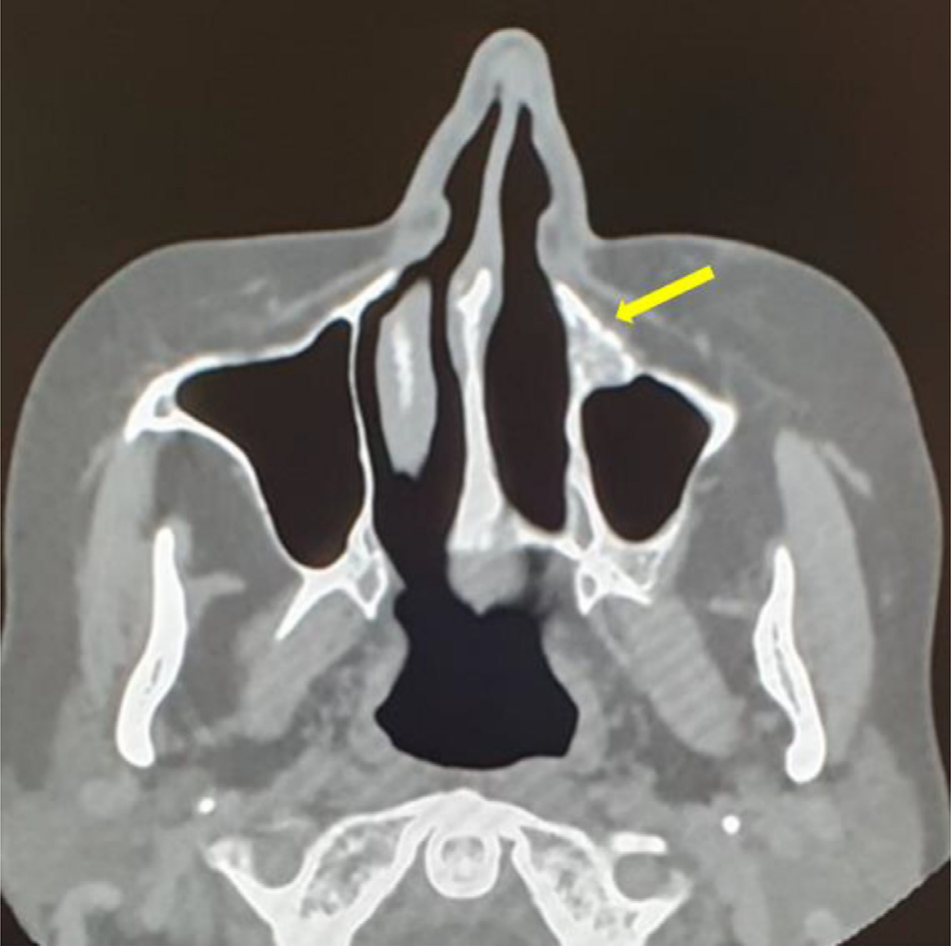
Follow-up CT showed reduction in lesion size. Presence of small residual neoplastic tissue at the level of the antero-medial region of the left maxillary sinus was detected, evidence of therapeutic efficacy.

Three months after the start of the therapy, the follow-up was performed by CT examination (Fig. 8) which demonstrated a significant reduction in the size of the lesion with a small residual disease occupying the antero-medial portion of the maxillary sinus, confirming the therapeutic efficacy.

## Discussion

In our case, CT images suggested the presence of a neoplastic mass that showed inhomogenous contrast-enhancement, erosion of the left maxillary sinus floor and extension to the mouth. MRI is very helpful to evaluate the soft tissue components and soft tissue extent of the neoplasm [3,12]. The MRI scan we performed, confirmed the presence of only one lesion extending from the left maxillary sinus to masticator space with erosion of the left alveolar process. Many CT and MRI features are not specific and it is important to find specific imaging characteristics to differentiate a plasma cells tumor, such as EMP, from other more common neoplasms of the sinonasal tract, including squamous cell carcinoma (SCC) and non-Hodgkin lymphoma (NHL), because therapy of these diseases is different [2,3]. In the case we presented, DWI sequence suggested the diagnosis of EMP. ADC values of the lesions we presented, were lower than ADC values that are associated with SCC. In fact some studies have shown that ADC values of SCC (0.96 × 10^−3^ ± 0.24 × 10^−3^ mm^2^/s) are higher than those of NHL (0.60 × 10^−3^ ± 0.33 × 10^−3^ mm^2^/s) and EMP (0.66 × 10^−3^ ± 0.16 × 10^−3^ mm^2^/s) [2,3,12]. ADC values of EMP are quite similar to ADC values that are associated with NHL, both tumors are isointense on T1-weighted images and slightly hyperintense on T2-weighted images, but EMP is more heterogenous (both before and after contrast medium administration) than NHL [2,3]. However it is not easy to differentiate EMP from other neoplasms, such as SCC and NHL without histopathological examination [2].

Another pathological entity that must be considered in the differential diagnosis, although rare, is the localization of melanoma [13]. In addition to the T1 hyperintensity of the melanoma lesion, a finding that is not found in EMP (which has medium-low signal in T1), the ADC value is slightly higher (0.87 × 10^−3^mm^2^/s) compared to the value we found. The spectroscopic study (MRS) did not contribute to reaching the correct diagnosis.

In our clinical case, biopsy was necessary to confirm the diagnosis, because it allowed to find cells positive for CD138, that is a specific surface antigen for plasma cells in the bone marrow and multiple myeloma cells [10].

## Conclusions

Diagnosis of EMP requires a multidisciplinary approach and finding specific imaging features is important for differential diagnosis. In the case we presented, DWI proved to be useful to suggest the diagnosis of EMP, such that it can be considered as an imaging marker for differential diagnosis. Therefore we recommend to include DWI sequence in MRI protocol in the suspicion of expansive process of the maxillary sinus and buccal cavity. Other imaging features, such as tumor heterogeneity and avid enhancement can be assessed, in association with ADC values, to distinguish EMP from other sinonasal neoplasms.

## Patient consent

Written informed consent was obtained from the patient.
